# Digital Phenotyping Data to Predict Symptom Improvement and Mental Health App Personalization in College Students: Prospective Validation of a Predictive Model

**DOI:** 10.2196/39258

**Published:** 2023-02-09

**Authors:** Danielle Currey, John Torous

**Affiliations:** 1 Department of Psychiatry Beth Israel Deaconess Medical Center Harvard Medical School Boston, MA United States; 2 Case Western Reserve University School of Medicine Cleveland, OH United States

**Keywords:** mHealth, mental health, smartphones, phenotype, symptom, college, students, young adults, responsive, personalized, app, application, intervention, effectiveness, protocol, model, digital, engagement, algorithm, usage

## Abstract

**Background:**

Mental health apps offer a transformative means to increase access to scalable evidence-based care for college students. Yet low rates of engagement currently preclude the effectiveness of these apps. One promising solution is to make these apps more responsive and personalized through digital phenotyping methods able to predict symptoms and offer tailored interventions.

**Objective:**

Following our protocol and using the exact model shared in that paper, our primary aim in this study is to assess the prospective validity of mental health symptom prediction using the mindLAMP app through a replication study. We also explored secondary aims around app intervention personalization and correlations of engagement with the Technology Acceptance Model (TAM) and Digital Working Alliance Inventory scale in the context of automating the study.

**Methods:**

The study was 28 days in duration and followed the published protocol, with participants collecting digital phenotyping data and being offered optional scheduled and algorithm-recommended app interventions. Study compensation was tied to the completion of weekly surveys and was not otherwise tied to engagement or use of the app.

**Results:**

The data from 67 participants were used in this analysis. The area under the curve values for the symptom prediction model ranged from 0.58 for the UCLA Loneliness Scale to 0.71 for the Patient Health Questionnaire-9. Engagement with the scheduled app interventions was high, with a study mean of 73%, but few participants engaged with the optional recommended interventions. The perceived utility of the app in the TAM was higher (*P*=.01) among those completing at least one recommended intervention.

**Conclusions:**

Our results suggest how digital phenotyping methods can be used to create generalizable models that may help create more personalized and engaging mental health apps. Automating studies is feasible, and our results suggest targets to increase engagement in future studies.

**International Registered Report Identifier (IRRID):**

RR2-10.2196/37954

## Introduction

Digital mental health solutions, especially smartphone apps, are recognized as a scalable means to increase access to care. With the rising crisis around youth mental health [1], compounded by the pandemic and the chronic lack of adequate services on college campuses [2], college mental health is a prime application for such apps. Yet, the impact of these apps to date has been minimal, not because apps are ineffective but rather because engagement is often low [3,4].

This study explores methods for improving engagement, with a focus on personalization. While many apps can deliver evidence-based content, there is evidence that students want apps to be more tailored to their personalized needs [5]. This personalization requires first predicting individual symptom changes and second responding with appropriate interventions. Focusing on the first step, digital phenotyping can advance prediction by using smartphone sensors to derive behavioral features (eg, sleep, steps) that can be incorporated into models. While prior research on digital phenotyping has proposed models for college student mental health [6], these models have never been prospectively validated. Following our published protocol [7], in this study, we prospectively validated a digital phenotyping symptom prediction model for college students. To our knowledge, this is the first digital phenotyping algorithm to be prospectively validated.

Our study app, mindLAMP [8,9], provides a useful platform for this research, as it offers both digital phenotyping as well as a suite of cognitive therapy–based exercises and skills that participants can access on demand or as scheduled. This enables mindLAMP to be a responsive app and use digital phenotyping–derived symptom prediction to help recommend individual app activities for a student. While mindLAMP did not offer just-in-time adaptive interventions in this study, we piloted the feasibility of responsive interventions using passive data features as a secondary aim. Such an exploration of feasibility is important, as few other apps have used digital phenotyping data in predictive models. A recent review of just-in-time adaptive intervention apps found that 71% of these apps use only self-report (ie, not digital phenotyping data), and considering all apps, only 3.6% of all measurements involved sensor and device analytics [10].

This same report that noted how little is known about the role of sensor data in just-in-time adaptive interventions also discussed how there is a lack of research on the theoretical basis and mechanism of action for how these apps drive engagement or outcomes. Theoretical models like the Technology Acceptance Model (TAM) [11] can help elucidate factors associated with app engagement but, to date, have been rarely used in this research. Specifically, when using methods like digital phenotyping it remains unknown if these efforts toward increased personalization increase positive attitudes about the app, make the app more useful, or both. Other factors beyond those explored in the TAM, such as alliance and connection to the app (measured with the Digital Working Alliance Inventory [DWAI] [12]) are also important to explore.

While in prior research studies we have shown that students can engage with therapeutic activities within mindLAMP [13], our use of the app activities in this study was to assess the recommender model. Building off our prior work with the mindLAMP app [8,9], in this study, we sought to validate our digital phenotyping symptom prediction model as a primary goal while secondarily assessing the feasibility of an app activity recommender model and the correlations with elements of the TAM and DWAI.

## Methods

### Ethics Approval

This study was approved by the Beth Israel Deaconess Medical Center institutional review board (protocol 2020P000310).

### Study

The protocol for this paper was published in *JMIR Research Protocols* [7]. This study used the open source mindLAMP app developed by the Digital Psychiatry lab at Beth Israel Deaconess Medical Center to collect survey and sensor data [8,9]. Briefly, the study recruited undergraduate participants via Reddit to complete a screener survey that was required to participate. The following inclusion criteria were used: students must be 18 years or older, score 14 or higher on the Perceived Stress Scale (PSS) [14], be enrolled as an undergraduate for the duration of the study, own a smartphone able to run mindLAMP, be able to sign informed consent, and pass a run-in period (outlined below). A link to the REDCap screener survey was posted on 73 different college Reddit web pages. Participants that were screened completed an informed consent quiz, which then automatically generated a mindLAMP account log-in.

Participants entered a 3-day run-in period, and if they completed the requirements (daily surveys and GPS passive data coverage checks), they were moved into the 28-day enrollment period. Passive data coverage was estimated as the percent of 10-minute intervals that had at least one GPS data point collected. If coverage was greater than 20%, participants were able to continue into the enrollment period. It is important to note that some level of missingness is expected in digital phenotyping work. We did not perform any imputation of passive data in our analysis.

During the enrollment phase of the study, participants had a feed with activities scheduled each day (eg, mindfulness or gratitude journaling) and completed longer weekly surveys (which included the Patient Health Questionnaire-9 [PHQ-9] [15], Generalized Anxiety Disorder-7 [GAD-7] [16,17], PSS [14], UCLA Loneliness Survey [18], Pittsburgh Sleep Quality Index [PSQI] [19], DWAI [12], and TAM-related questions [11]) each week. Participants were compensated for completing weekly surveys at weeks 1, 3, and 4 (US $50 in total) but not for engaging with any interventions of the coverage of digital phenotyping captured through their smartphones. However, participants were warned via email if they did not complete any activities for 3 days and were discontinued from the study if they still had not completed any activities after 5 consecutive days. In addition to these activities, one-third of participants received emails from a “research assistant” (emails were automated but signed with a researcher’s name) encouraging them to complete an additional activity based on their data. Another one-third of participants were sent an activity suggestion from the study bot “Marvin.” The final one-third of the participants did not receive any additional activity suggestions. As explained in the protocol [7], participants were sequentially assigned to each of the three groups.

This study is important because it was a successful iteration of a fully automated study. All aspects of the study, including enrollment, data quality checks, payment, and activity suggestions and scheduling, were automated by Python study workers. For further details on the study, please refer to the protocol [7]. We would like to note that there were no deviations from the protocol. The code for the automated workers is open source and available on GitHub [20].

### Enrollment

**Figure 1:**
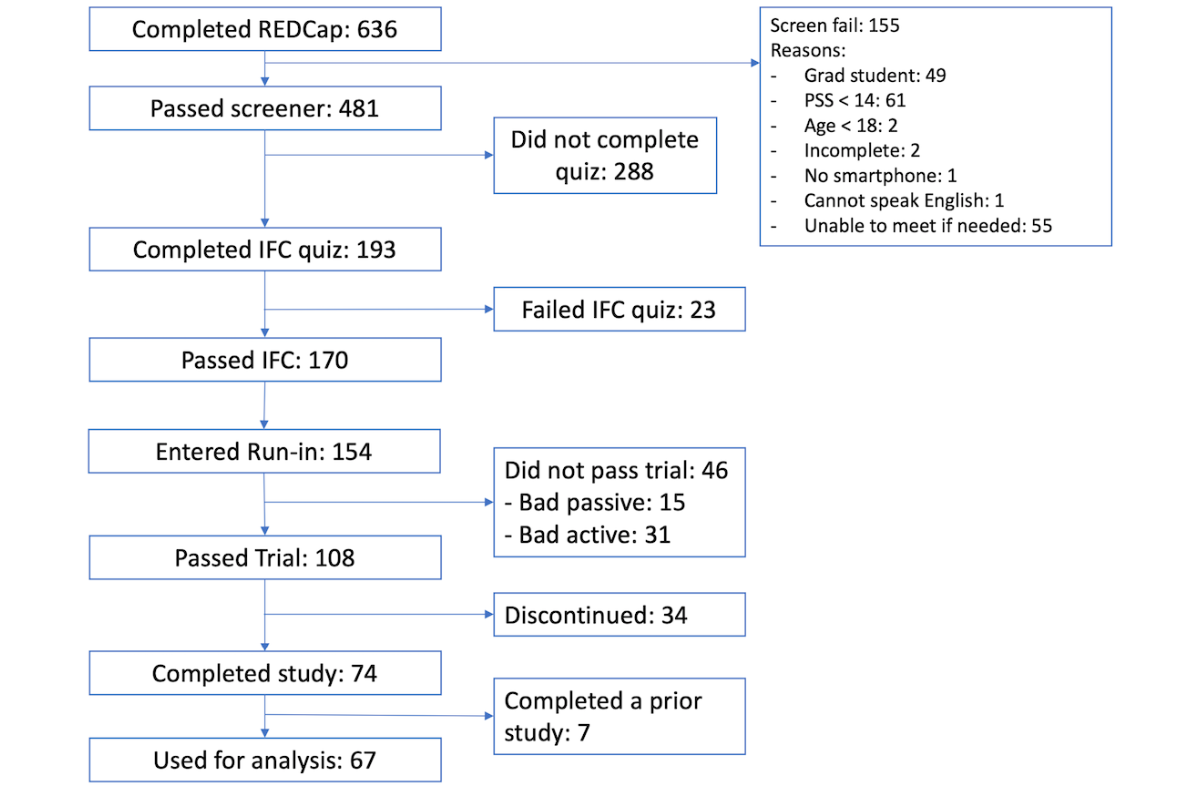
Flowchart showing counts of participants at different stages in the study. IFC: informed consent; PSS: Perceived Stress Scale.

A total of 67 participants were used for this analysis. The number of participants that completed each phase is shown in Figure 1. A total of 636 participants filled out the initial screening survey, 481 of whom met the study requirements outlined above; 170 of these participants completed and passed a required informed consent quiz designed to ensure participants understood the expectations of the study. Of these, 154 created mindLAMP accounts and entered the run-in period. A total of 46 of these participants did not make it through the run-in because they either did not complete all the daily surveys (bad active data) or did not meet the passive data coverage requirement (bad passive data). Some participants had both “bad” active and passive data and were counted under “bad active.” Of the 108 participants that entered the enrollment period of the study, 34 were discontinued after not completing any activities in the app for 5 consecutive days. A total of 74 participants completed the study.

Some participants were excluded from the analysis. First, the initial screener survey had an error where 3 participants who reported being unable to meet if needed were allowed to complete the informed consent. These participants are shown in Figure 1 under “unable to meet if needed” and were excluded from the analysis (the numbers do not sum to the total for screen-fail as some people were excluded for multiple reasons). Second, after retrospectively reviewing the REDCap screener survey entries, it seems that some participants filled out the survey multiple times, changing their responses to be included in the study. One participant changed their status from “graduate” to “undergraduate,” and 4 other participants changed their responses to the PSS survey to increase their scores by double or more. While stress does fluctuate over time, such a large change over only about 10 minutes is unlikely. These 5 participants are not shown in Figure 1 and were excluded from the analysis.

Finally, 7 of the 74 participants that completed the study previously participated in the College Study (V1 or V2), so they were excluded from the analysis to ensure the sample used for model testing was completely distinct from the training and testing sets. The demographics of participants used in the analysis are outlined in Table 1. Of the 67 participants used for analysis, 52 used iOS phones and 15 used Android phones. Participants had a mean age of 20 (SD 2) years.

**Table 1:** Demographic information.

Race	Female, n	Male, n	Nonbinary, n	Total, n
Asian	11	6	1	19^a^
African American	2	0	0	2
Latinx	7	1	0	8
White	24	8	6	38
Total	44	15	7	67

^a^One Asian student marked their gender as “prefer not to say.”

### Prospective Model

The model reported in the protocol [7] was prospectively tested on this data set. The 67 participants used for testing have not previously participated in a College Study and were completely distinct from the testing and validation training sets.

### Groupwise Analysis

As a secondary outcome, the Python scipy.stats [21] was used to perform ANOVA tests on each feature to determine if differences existed between the groups receiving suggestions from a digital navigator or a bot, or receiving no suggestions. We also used the scipy.stats module to perform *t* tests comparing the group that completed the suggested activities to the group that did not. Here, we examined the TAM [11] and DWAI [12,22] questions from the weekly survey to investigate the relationships between attitudes toward the app and behavior. We did not aim to validate the TAM but rather to explore questions related to engagement. *P* values were corrected using the Hochberg method via the statsmodels.stats.multitest.multipletests module in Python [23].

## Results

**Figure 2:**
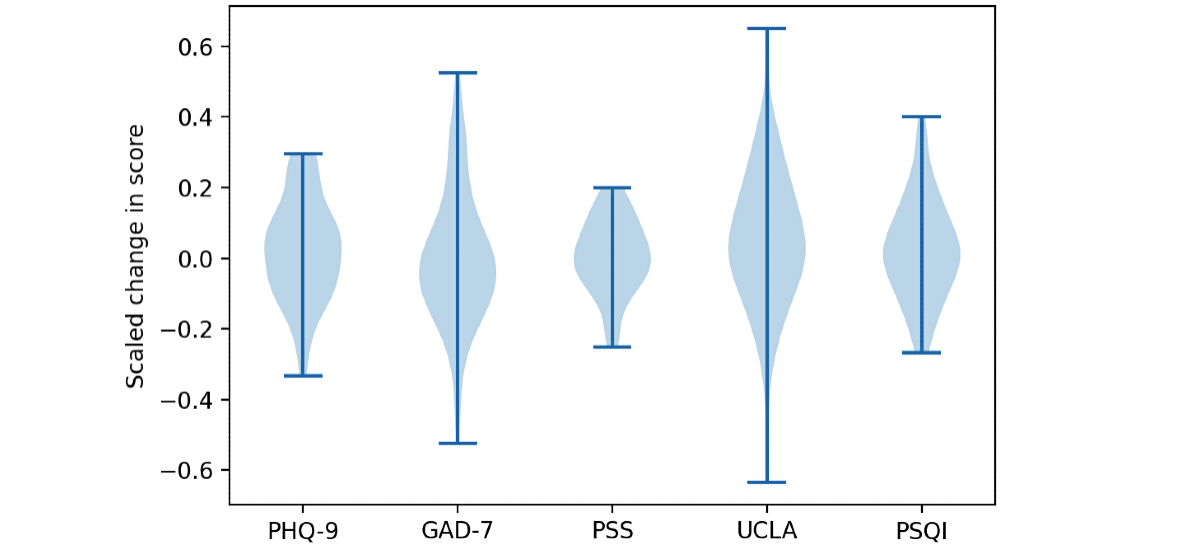
Violin plots showing the change in scores for each survey scaled by the total possible score (PHQ-9: 27, GAD-7: 21, PSS: 40, UCLA: 60, PSQI: 15). Score differences are plotted as starting score – ending score, so that positive values indicate improvement. GAD-7: Generalized Anxiety Disorder-7; PHQ-9: Patient Health Questionnaire-9; PSQI: Pittsburgh Sleep Quality Index; PSS: Perceived Stress Scale; UCLA: UCLA Loneliness Survey.

The primary goal of this study was to prospectively validate a model to predict whether a participant would improve over the course of the study given the average of each of their passive and active data features. The change in score by participant (scaled by the number of points in the survey) is shown in Figure 2. Except for the GAD-7, which showed a slight average increase in the score (0.0025), participants’ scores on average decreased. The mean and SD for the weekly survey scores across students are listed in Table 2.

**Table 2:** Mean and SD of weekly survey scores across students.

Survey	Scores, mean (SD)
Patient Health Questionnaire-9	7.55 (4.45)
Generalized Anxiety Disorder-7	6.49 (3.90)
Perceived Stress Scale	18.04 (6.33)
UCLA Loneliness Survey	15.56 (15.05)
Pittsburgh Sleep Quality Index	5.30 (2.77)

Area under the curve (AUC) values range from 0.58 (UCLA Loneliness) to 0.71 (PHQ-9). Specifically, the AUC values were 0.71 for PHQ-9, 0.60 for GAD-7, 0.68 for PSS, 0.58 for UCLA Loneliness, and 0.60 for PSQI.

There were no significant differences in the survey features (*P*>.05), the number of activities completed (*P*>.99), or the percentage of module activities completed between the three groups (*P*>.99). Moreover, the changes in the PHQ-9 and GAD-7 scores were not significantly different between the three groups (*P*=.42 and *P*=.72, respectively). Despite the relatively high completion of scheduled module activities (73% on average), few participants completed the optional activities suggested by the recommendation algorithm. Over half (24/41, 59%) of the participants who received suggestions completed at least one activity. Two-sided *t* tests were performed to compare the DWAI and TAM question scores between the group that completed at least one activity and the group that did not complete any of the optional activities. These *P* values can be found in Table 3.

The differences between the groups were in the questions about the perceived usefulness of the app. There were no significant correlations between the magnitude of the PHQ-9 or GAD-7 scores’ improvement and the DWAI score, the number of activities completed, or the percentage of assigned activities completed. Finally, there were no significant correlations between the average PHQ-9 and GAD-7 scores and DWAI or TAM scores.

**Table 3:** *P* values for the t test comparison between participants that did and did not complete at least one suggested activity.

Question			Component of TAM^a^ model		*P* value
**DWAI^b^**					
	Total score	N/A^c^		.77	
	I agree that the tasks within the app are important for my goals.	Attitude toward using		.07	
	I believe the app tasks will help me to address my problems.	Attitude toward using		.07	
	I trust the app to guide me toward my personal goals.	Attitude toward using		.24	
	The app encourages me to accomplish tasks and make progress.	Attitude toward using		.31	
	The app is easy to use and operate.	Perceived ease of use		.48	
	The app supports me to overcome challenges.	Perceived usefulness		.39	
**TAM**					
	Total score	N/A		.07	
	I want to use the app daily.	Behavioral intention to use		.27	
	I would want to use it after the study ends.	Behavioral intention to use		.18	
	The app allows me to easily manage my mental health.	Perceived usefulness		.31	
	The app makes me better informed of my mental health.	Perceived usefulness		*.01^d^*	
	The app provides me with valuable information or skills.	Perceived usefulness		*.01*	

^a^TAM: Technology Acceptance Model.

^b^DWAI: Digital Working Alliance Inventory.

^c^N/A: not applicable.

^d^Italicized *P* values indicate values less than .05.

## Discussion

### Principal Findings

The primary outcome, validation of the symptom prediction model, demonstrated overall success with an AUC of 0.71 for change in depression symptoms measured by the PHQ-9, which is similar to the results from prior studies referenced in the protocol [7]. This prospective validation indicates that such models may be able to generalize across samples and thus be applicable to a broad range of college students.

Our results also explored engagement with apps as secondary outcomes. Overall engagement with assigned tasks in the app was 73%. Participants who completed at least one of the optional recommended activities scored differently on certain TAM questions. In particular, the usefulness questions around the belief that the app provides some helpful/valuable information differed, indicating that these types of attitudes toward the utility of the app may be necessary for participants to engage. However, our data set is small and not powered for these outcomes, so further work is needed to explore questions around which participants are best suited to benefit from using digital mental health apps.

Deploying the app recommendation algorithm demonstrated feasibility but did not in itself change engagement. This was likely due to our study not being designed or powered to change engagement but rather to replicate the prediction algorithm and demonstrate the feasibility of using it to automate recommendations.

### Strengths and Limitations

Our study was limited by the sample size and the fact that all participants were college students. First, a larger sample size would provide better training and testing data, and would allow for improvements in the symptom prediction model. Moreover, the small number of participants in each engagement subgroup (about 20 per group) makes it difficult to compare them. In the future, larger sample sizes should be recruited to further investigate the difference between interacting with a digital navigator and not.

Second, although there is a high level of need in the college student population for mental health resources, using college students can also be considered a limitation of this study, as college mental health may not be representative of the broader experience for the general population. Moreover, the way that college-aged students interact with their phones may be different from the rest of the population, making phenotyping methods difficult to transfer to other groups. Future work should explore generalization across age and other demographic groups and seek a more gender-balanced sample than ours. Related work suggests that bias across races and different ethnic groups may be low [24], but this needs to be assessed in future work. Qualitative results around app engagement with mindLAMP, as we have done in the past with college students [25], will be important to explore with new populations. More work is also necessary to validate symptom prediction models in different populations, especially those with lower digital health literacy or students with different backgrounds than those featured in our sample. Still, given the high degree of mental health needs in this population, our results can support future efforts to personalize apps toward delivering more tailored care.

### Future Directions

While our activity tailoring algorithm did not drive engagement, overall engagement was high in our study. Weekly therapeutic module completion was high despite these activities not being required or compensated. We did not assess the reasons for this higher engagement, but perhaps by scheduling activities in the feed, participants felt that they should complete these daily activities, while the additional activities recommended by the activity recommendation algorithm were explicitly provided as suggestions. Adding the recommendation algorithm activity suggestions into the feed is a simple next step to assess in future studies. Moreover, since our work suggests that attitudes around app usefulness contribute to engagement, future work should also explore whether participant attitudes can be changed. If the belief that the app is helpful is the key to engagement, then focusing on changing this attitude may be the key to reaching technology-resistant participants. We also note that our results around engagement were secondary outcomes, and our analysis involved a first-pass overview with assumptions such as the underlying data being normally distributed. As the field seeks to better operationalize measures of engagement [26,27], digital phenotyping metrics like those featured in this paper may play a future role [28,29].

Our results around overall study recruitment and retention are also important for planning and powering such future studies [30]. We were able to obtain a high-quality data set, but this required recruitment of almost three times our goal due to the loss of participants through the run-in period and the requirement for a baseline level of participation. In addition, the fact that we had at least five participants providing false information to enter the study underscores the challenge of web-based recruitment the field is now growing aware of [17]. We expect this likely impacts all web-based studies and hope that, by calling attention here, others will also carefully consider who is enrolled in their digital health research.

### Conclusion

Overall, this study presents evidence that a digital phenotyping symptom prediction model can prospectively generalize to a new population of college students. The success of the automated study protocol holds promise for being able to efficiently run even larger studies in the future, and the results around activity tailoring suggest areas for future improvement.

## Abbreviations

AUC: area under the curve
DWAI: Digital Working Alliance Inventory
GAD-7: Generalized Anxiety Disorder-7
PHQ-9: Patient Health Questionnaire-9
PSQI: Pittsburgh Sleep Quality Index
PSS: Perceived Stress Scale
TAM: Technology Acceptance Model

## Data Availability

The code for the automated workers is open source and is available on our GitHub. Data from this study is not available given the personally identifiable nature of the information.
